# How Do We Achieve Impactful Patient and Public Involvement and Engagement in Statistical Methodology Research? A Qualitative Research Protocol

**DOI:** 10.1111/hex.70777

**Published:** 2026-08-06

**Authors:** Julie Roberts, Samina Begum, Beatriz Goulao, Nicola Mackintosh, Michelle O'Reilly, Christopher Newby, Laura J. Gray

**Affiliations:** ^1^ Division of Public Health and Epidemiology, School of Medical Sciences University of Leicester Leicester UK; ^2^ Public Contributor and Co‐Author University of Leicester Leicester UK; ^3^ School of Health & Wellbeing University of Glasgow Glasgow UK; ^4^ School of Criminology, Sociology & Social Policy, School of Psychology & Vision Science University of Leicester Leicester UK; ^5^ Leicestershire Partnership NHS Trust Leicester UK; ^6^ Faculty of Medicine and Health Sciences University of Nottingham Nottingham UK; ^7^ NIHR Applied Research Collaboration East Midlands University of Leicester Leicester UK; ^8^ NIHR Leicester Biomedical Research Centre University of Leicester Leicester UK; ^9^ Leicester British Heart Foundation Centre of Research Excellence University of Leicester Leicester UK

**Keywords:** composite case studies, co‐production, guidance, patient and public involvement and engagement, protocol, qualitative research, statistical methodology

## Abstract

**Background:**

Patient and public involvement and engagement (PPIE) is vital for good‐quality health research. However, PPIE is more commonly practised in some types of research than others. Statistical methodology research aims to improve the way we design studies and analyse data. It can be particularly challenging to involve members of the public and patients in this type of research because the work can be technical and difficult to explain. Consequently, PPIE work typically requires considerable time and resources. Our previous research suggests that most statistical methodologists do not feel confident in carrying out PPIE for statistical methodology research and would benefit from access to new guidance and training.

**Methods:**

This qualitative, ethnographic study will combine observations and interviews to generate new insights into what works well, and what the challenges are, when involving the public in statistical methodology research. Data will be analysed using the template approach and presented in composite case studies, generating an interactive guidance document for researchers and PPIE groups to support the practice of impactful PPIE in statistical methodology research.

**Dissemination:**

We will hold a virtual launch event for the co‐produced guidance and case studies. These will be made freely available on a dedicated website and *Learning for Involvement*, and highlighted via social media. The work will be presented at key conferences and published in open‐access peer‐reviewed journals. Findings will also be shared in educational resources to influence the next generation of researchers.

**Patient or Public Contribution:**

The study design has been informed by the perspectives of public co‐applicants on the grant. The research team will work alongside a public advisory group (PAG) consisting of six public contributors with extensive PPIE experience. The PAG will be chaired by Begum, a public co‐applicant. We will recruit three more public contributors to take part in online workshops to develop the main output of the study.

## 1 Introduction

Public involvement in research is ‘an active partnership between patients, carers and members of the public with researchers that influences and shapes research’ [1], no pagination]. The value of PPIE is underpinned by a moral and democratic view of the necessity of representation of voices from populations who may ultimately be affected by the research findings [2]. There is growing international interest in patient and public involvement and engagement (PPIE) [3]. PPIE is, however, well embedded in applied health care research in the United Kingdom (https://www.amrc.org.uk/guidance-and-tools-for-public-involvement), with PPIE a requirement of some UK research funders.

Statistical methods are fundamental to good‐quality health research and crucial for informing evidence‐based clinical practice and policies. Statistical methodology research involves the development, evaluation or comparison of statistical methods for the design or analysis of research studies. Using the correct statistical methods and finding novel ways to analyse data can have far‐reaching relevance to the study of different health conditions, therapies and clinical decision‐making processes. The impact of statistical methodology research can be wide‐reaching—such as reducing the sample size required to answer a specific research question, enabling more precise estimation of results or preventing bias, or preventing clinicians from drawing erroneous conclusions due to an incorrect analysis approach.

Although challenges remain, there are excellent examples of how patients and the public have become meaningful partners in applied health research, ensuring that the research is of good quality, understandable and relevant to those that it impacts. However, there are fewer examples of impactful PPIE in statistical methodology research compared to applied health research [4]. Yet, case studies suggest that PPI can have benefits, including improved model structures and identification of data issues [5]. The Medical Research Council (MRC) published a review of public involvement in non‐clinical health and biomedical research, within which statistical methodology research falls. The review highlights the equal importance of public involvement in both clinical and non‐clinical health research, and provides recommendations for improving practice [6].

However, there is uncertainty among statistical methodologists about the feasibility and relevance of PPIE [5]. It can be particularly challenging to involve members of the public and patients in statistical methodology research because the work can be technical and difficult to explain, which means that it often requires considerable time and resources to conduct PPIE. We previously conducted a survey of statistical methodologists, and over 80% did not feel confident in carrying out PPIE for statistical methodology research. When asked what would make them feel more confident about undertaking PPIE, training, guidance and case studies were the top responses. Many statisticians are willing to involve members of the public in their research, but simply do not know where to start. A recent survey of statisticians found that over 80% of respondents did not feel confident in carrying out PPIE for statistical methodology research [7]. There is currently no guidance available about how to involve the public in this field of research.

This study adopts an innovative, qualitative approach to investigate how we can achieve impactful PPIE in statistical methodology research. Impact is defined broadly to include both how the research process is changed by PPIE and impacts for individuals that might be harder to measure but are nonetheless significant, including impacts on understanding, values and skills [8, 9].

## 2 Study Aim and Objective

The aim of this study is to empower and equip statistical methodology researchers to include meaningful, impactful public involvement in their research.

The primary objective of the research is to develop guidance to support researchers and public contributors to conduct impactful PPIE to inform statistical methodology research.

## 3 Methods

This study builds on our previous work in this field, including an established PPIE group, workshops and co‐produced resources (PPISmart). This qualitative, ethnographic study will bring together observation data from different groups with interview and document analysis data to develop composite case studies [10] to guide future PPIE activities. The project will be conducted between October 2025 and May 2027. The research will be conducted by a multi‐disciplinary team of statistical methodologists and qualitative methodologists, all with expertise in health‐related research.

### 3.1 Research Governance

Drawing on our combined knowledge of the field, we have identified and recruited an expert advisory group of four academics with relevant expertise and experience of PPI in different types of research, including statistical methodology research. The advisory group will meet three times over the lifetime of the project to provide guidance on emergent issues and themes.

Six members were recruited to a public advisory group (PAG). We advertised via dedicated web directories of PPI opportunities (People in Research; Shaping Our Lives). People submitted expressions of interest by completing a short form detailing the length of experience of PPI, providing examples of research that they had been involved in and writing what interested them about the project. Members were selected by the public co‐applicant and the researcher on the team, aiming for diversity of experience, age and ethnic background. Short informal telephone calls were used to confirm interest and appointment to the project. The PAG will be chaired by a public co‐applicant on the project team and will meet at least six times over the lifetime of the project to provide guidance on emergent issues and themes. Meetings will be at key time points, including at the start, mid‐point and end of data collection, during analysis and dissemination planning.

Final decisions will rest with the research team, taking into account the expert advice of the advisory group and the PAG.

Ethical approval has been received from the General Research Ethics Committee, University of Leicester (ID 4345).

### 3.2 Data Collection

#### 3.2.1 Observations of PPIE Groups Involved in Statistical Methodology Research

Non‐participant observation of PPIE meetings will provide first‐hand insights into how statisticians involve public contributors, communicate complex statistical methodological concepts and respond to public contributors' input. Observations will also capture the strategies used to balance power relations and enable participation and inclusion within PPIE.

We will purposefully select PPIE groups in the United Kingdom, working across different types of statistical methodologies, aiming for a minimum of six groups and observing one to two meetings per group where statistical methodology is being discussed. We expect this to provide rich descriptions of diverse practices and methodologies.

We will target holders of NIHR fellowships undertaking relevant research and holders of MRC/NIHR Better Methods for Better Research awards. We will also seek expressions of interest through a number of channels, including our previous survey and workshop participants, studies identified in a scoping review [4, 7, 11], targeted invitations to those holding NIHR/MRC funding related to statistical methodology and through mailing lists and social media spaces that target statisticians. This recruitment approach reflects the current landscape in which statistical methodologists are often prompted to undertake PPI by funder requirements, whereas mailing lists and social media have potential to reach beyond these funded projects.

Access to PPIE meetings for observation will be agreed with the researcher or PPI group chair convening the group. We will provide study information to leads, who will discuss participation with their group members. Where groups are in agreement on participation, leads will circulate study information sheets to potential meeting attendees at least 3 days before the meeting(s) to be observed. It will not be possible to receive individual written consent during observations without significant disruption to the work of the PPIE groups. However, the researcher will introduce themself and the project at the start of meetings, remind attendees about the research and that observation is taking place, and invite questions. Participants will be reminded of their right to withdraw without providing a reason. Participation in the meeting will constitute consent to take part in the research. The researcher will make themselves known to any attendees joining the meeting late, verbally when meeting in person or using the chat when meeting online. The researcher will thank participants at the end of the meeting and indicate willingness to answer further questions about the research.

Observations will be conducted by an experienced qualitative researcher. An observation guide (see the supporting information) has been designed with public consultation, and guided by the research questions and with reference to available standards: *UK Standards for Public Involvement* [12]; the Public Involvement Impact Assessment Framework (PIIAF) [13]; and the CUBE PPI evaluation framework [14]. Observations will focus on the values underpinning PPIE (e.g., the purpose and value of PPIE [substantive and normative], norms of the group and ways of working together), specific approaches and methods of involving members of the public in statistical methodology research (e.g., details of presentations, tasks and discussion), how power differentials are managed within public contributors and between contributors and researchers, and any evidence of the impact of PPIE on the research. The researcher will make brief notes during observed meetings. As soon as possible after the meeting, the researcher will elaborate fieldnotes in audio form. These will be anonymised at transcription where necessary and allocated a unique identifier. Documents relating to the group and the meeting will also be collected, including terms of reference, agendas, minutes and reports, adding to the fieldnotes and, in some cases, providing wider context. Where a PPIE group meets in person, the researcher will attend in person. Online meetings will be observed remotely.

#### 3.2.2 Individual Semi‐Structured Interviews With Researchers and Public Contributors

We will interview one Principal Investigator or PPIE group lead from each group that we observe (six interviews). We will select 3–4 groups that have been observed and invite additional research staff and public contributors who were part of the observations (estimate 30 interviews). To allow us to hear from people with relevant experience from completed projects or from groups that do not wish to take part as a collective, we will also advertise for up to 10 individual participants via the website People in Research, through statistics professional mail lists and using contact lists from previous research in this topic area (estimate 10 interviews). Participants will be purposively selected to maximise the diversity of career stage, extent of PPI experience, type of methodological project and geographical location.

Interviewees will receive study information and a consent form at least 24 hours before an interview. Participants will have three options for returning consent forms to ensure that processes are accessible and inclusive:

i. Signing paper copies at the interview (in‐person interviews only).
ii. Returning the consent form by email (where participants have access to a printer/scanner).
iii. Verbally agreeing to each item on the consent form at the start of the interview. Verbal consent will be audio‐recorded and transcribed.

Interviews will be conducted in person or via MS Teams by an experienced qualitative researcher. Interviews will take up to 90 minutes of participants' time, including checking eligibility and receiving consent. We will minimise the burden to participants by offering flexibility over the time and place of the interview. Interviewees will receive £50 in shopping vouchers in thanks for their time and contribution to the research. Topic guides (see the supporting information) for researchers and public contributors have been designed based on public consultation and with reference to available standards (as above). Interviews will focus on values, approaches, power differentials and impacts. Basic demographic information will be collected (refer to topic guides).

Interviews will be recorded within MS Teams. Only audio will be transcribed. Data will be transferred securely to a professional transcriber with a confidentiality agreement with the University. Data will be anonymised at transcription and allocated a unique identifier.

### 3.3 Data Analysis

Analysis will follow a template approach to identify and cluster core issues and to diagrammatically map the relationships between them [15]. Template analysis benefits from a blend of inductive (data‐driven) and deductive (theory‐driven) coding processes to create an insightful template to represent the data and to identify the relationships between themes [16, 17]. Deductive analysis will be guided by the research questions and existing models of PPIE (e.g., [13, 14]). We will draw on rapid ethnography methods, including regular debriefs of the field researchers with the research team and the production of summaries of data per study, organised by developing themes. We will use merging and mapping to integrate the findings from the observations, documentary analysis and interviews to textually and visually represent the core issues [15]. Data types will be treated equivalently, and we will explore synergies and differences.

The data analysis will inform development of a set of core good practice principles illustrated through composite case studies, where the details of each case are drawn from across the data set and do not reflect any single participating PPIE group. These will protect the anonymity of participants while illustrating best practice principles. These will be co‐produced by a group of researchers and public contributors working with the research team in three externally facilitated online workshops. Workshop participants will be recruited from people who have expressed an interest in the project at earlier stages, with calls for participation shared with professional networks and mailing lists to fill any gaps in expertise. Composite case studies will enable us to tell stories about what the data tell us. This approach involves bringing together data from several observations and interviews to tell a story framed as that of a single group or individual [18].

The workshops will (i) review the outputs of the research to date; (ii) co‐develop and refine the initial guidance; and (iii) develop the implementation plan. These workshops will include visual storytelling and live scribing. These approaches facilitate ‘reflective mirroring’, which encourages effective communication by fostering a culture of openness and collaboration between researchers and public contributors [19, 20]. Ownership, idea generation and consensus building are improved when live scribing is used in co‐production [20, 21]. To ensure that everyone feels comfortable contributing, we will offer one‐to‐one meetings before the workshops to answer any questions and to ensure that everyone feels prepared and understands how to use the functionality of Zoom/Teams. Breakout rooms will be used to foster conversation and to ensure that everyone is heard.

To finalise the guidance, we will hold an in‐person, stakeholder review meeting using the World Café approach [22]. Up to 40 participants (researchers and public contributors) will circulate around tables to collectively review each section of the draft guidance. Paper table cloths and marker pens will enable participants to draw or write as they discuss, and a scribe will capture feedback. Facilitators will be stationed at each table to provide continuity and feed in discussion points from previous groups. World Café approaches are especially useful when engaging large groups of participants from different backgrounds [23] and reduce researcher influence by encouraging participants to be actively empowered over the focus of conversations [24].

## 4 Ethics and Dissemination

The study has received ethical approval from the General Research Ethics Committee, University of Leicester. Informed consent will be obtained before any research takes place.

We recognise that carrying out observations of PPIE meetings may be sensitive and pose ethical challenges. Roberts, an experienced and sensitive qualitative researcher, will carry out the observations. At the start of each period of observation, the researcher will check in with PPIE members and research staff to obtain consent.

Confidentiality and anonymity will be assured to participants, unless there are clear and overriding reasons to do otherwise, for example, concerns about the safety of the interviewee or others. Exceptions to confidentiality will be outlined in the participant information sheets. The study team will adhere to GDPR principles at all times. Hard copies of consent forms will be kept in a locked filing cabinet, with access limited to the research team.

Identifying details will be removed at transcription and transcripts assigned a unique identifier. The data will be stored securely. If quotes from interviews are used in reports, they will be anonymised or pseudonymised.

Composite case studies will protect the identity of individuals and groups.

The co‐produced guidance will be made freely available on a dedicated website and *Learning for Involvement*, a collection of resources and training for PPIE hosted by the National Institute for Health and Care Research (NIHR) in the United Kingdom. The guidance will include composite case studies to demonstrate impactful PPIE and issues to avoid. We will hold a virtual launch event.

We will also share information via social media. We will present at key national and international conferences, and publish our findings in open‐access peer‐reviewed journals. We will record a webinar, which will be on our website. We will develop a session for a local MSc course in medical statistics on what PPIE is and the importance and relevance of PPIE for statistical methodology research. We will make the slides and accompanying notes freely available to encourage other similar courses to adopt them. A longer term aim is to develop online training courses and materials. Training early‐career researchers has been suggested as one way to build skills and expertise to adopt PPI in their ongoing research careers [25]. This project aims to contribute to this in the specific arena of statistical methodology research.

## 5 Public Contribution

This study is underpinned by extensive collaborations with members of the public, including an established PPIE group (PPI‐Smart, University of Leicester) and a previous workshop. The study was designed with the involvement of an established PPIE group, including two public contributors who became co‐investigators for the funding application. PPIE members raised in our planning meetings that public contributors may have health conditions, including mobility impairments, which can make it difficult to meet in person. We will be accommodating of contributors' needs, providing online and in‐person options where possible. When in‐person meetings are required, we will choose accessible venues.

One public co‐applicant will chair a PAG. The PAG consists of six public contributors with extensive PPIE experience between them, including involvement in statistical methods research and other types of data‐intensive research, and experience of producing training materials for research. We will recruit three more public contributors to take part in online workshops to develop the main output of the study.

## 6 Discussion

This study will produce the first guidance for PPIE in statistical methodology research. The literature, including our own previous research [7], suggests that PPIE in this field of research is underused and researchers lack confidence about how to conduct PPIE. The co‐produced guidance and resources resulting from this research will address this issue.

The guidance that we create will provide the research community with the tools to improve the way PPIE is done in statistical methodology research. This will lead to better research that benefits fully from the lived experiences of people who we eventually want to impact. We anticipate that our guidance will also help researchers in other technical fields that need good‐quality PPIE, such as data science and health economics.

The use of observation is a novel way to collect data about PPIE practice. It is much less commonly used than methods such as interviews and focus groups, or reflective pieces [26]. However, observation has been used successfully in this context, for example, in a study of PPIE meetings that moved online during Covid‐19 restrictions [27]. Observational methods are well suited to investigating complex, everyday practices. They are particularly useful where there are two or more groups of people who may experience the researched phenomenon differently, in this case, researchers and public contributors [28]. Interviews will supplement observation to ensure that we capture non‐observable aspects of PPIE practice, such as impacts of PPIE that happen in a different time or place to the observed meeting. Combining methods also allows triangulation of data about what works well; this is a gap in the current literature [29].

Composite narratives are able to represent multiple perspectives on a single scenario [30]. This will be important to ensure that researcher and public contributor voices are present in the narratives. Composite case studies also offer a way to maintain anonymity while remaining grounded in the data [10]. Relationships between researchers and public contributors are grounded in trust—at both an individual and societal level—and it is vital that participants can share their experiences without jeopardising this. Finally, composite narratives are rich, accessible and impactful ways to engage audiences with research findings [10, 18, 30] and will support uptake of the guidance across the research community.

Guidance for PPI has an important role to play in defining and promoting good practice internationally [31]. There is increasing research activity around producing guidance specific to particular areas of research, where experts in the field perceive unique challenges (e.g., [32, 33]). This study will meet a need identified in previous research [7] for guidance, training and case studies specific to statistical methodology research.

## Author Contributions

**Julie Roberts:** writing – original draft, project administration, methodology. **Samina Begum:** conceptualisation, funding acquisition, writing – review and editing, methodology. **Beatriz Goulao:** conceptualisation, funding acquisition, writing – review and editing, methodology. **Nicola Mackintosh:** conceptualisation, funding acquisition, writing – review and editing, project administration, methodology. **Michelle O'Reilly:** conceptualisation, funding acquisition, writing – review and editing, methodology. **Christopher Newby:** conceptualisation, funding acquisition, writing – review and editing, methodology. **Laura J. Gray:** conceptualisation, funding acquisition, writing – review and editing, methodology, project administration.

## Ethics Statement

The study received ethical approval from the University of Leicester General Ethics Committee on 9 October 2025 (Ref. 4345).

## Conflicts of Interest

The authors declare no conflicts of interest.

## Supporting information


Supporting File 1

Supporting File 2

Supporting File 3

## Data Availability

Data sharing is not applicable to this article as no data sets were generated or analysed during the current study.
