# Supplementary material for: How Do We Achieve Impactful Patient and Public Involvement and Engagement in Statistical Methodology Research? A Qualitative Research Protocol
Source: Health Expect. 2026 Aug 6;29(4):e70777. doi: 10.1111/hex.70777 (PMC13445566; doi:10.1111/hex.70777)
Supplement: Supplementary file 1 [file HEX-29-e70777-s001.docx]

# How do we achieve impactful patient and public involvement and engagement in statistical methodology research?

**Observation Guide**

# About the meeting

# Basic details about the meeting, time, venue (physical or online), number of people attending, research topics, agenda.

# 2: Values

# How is the purpose and value of PPIE communicated?

# How are norms of the group communicated?

# Any ground rules to make a good meeting

# 3: Approaches and Methods

# What are members of the public being asked to do?

# What strategies are used to communicate the research and key concepts?

# What activities and strategies are used to facilitate involvement?

# How do they work? E.g. Use of space, level of engagement, any prompts or props

# Who is doing this work?

# What seems to work well?

# What is challenging?

# How are different perspectives managed in the group?

# 4: Impacts

- What actions are agreed?
- Any impacts of PPIE reported to the meeting?
- Any evidence of record keeping?
- Details of any ongoing involvement
