# Supplementary material for: How Do We Achieve Impactful Patient and Public Involvement and Engagement in Statistical Methodology Research? A Qualitative Research Protocol
Source: Health Expect. 2026 Aug 6;29(4):e70777. doi: 10.1111/hex.70777 (PMC13445566; doi:10.1111/hex.70777)
Supplement: Supplementary file 2 [file HEX-29-e70777-s002.docx]

# How do we achieve impactful patient and public involvement and engagement in statistical methodology research?

**Topic Guide for public contributors**

**Welcome and Introduction**

Description of the project and the focus of the interview.

We are speaking with members of the public like you, who have been involved in statistical methodology research. By involved, we mean that you have advised or worked with a research team. Statistical methodology research is research that investigates the best statistical tools to collect, analyse and apply to a research question.

You are of course free to pause or stop the interview at any time. If there are any questions that you don’t want to answer, we can miss them out.

We are very mindful of the need to protect relationships between researchers and public contributors and we want you to feel able to speak freely. Your participation will be kept confidential. We may use direct quotations from your interview when we report the findings of the research but we will remove anything that could identify you. We will create composite case studies that will bring together experiences from different people, rather than reporting one person’s experience in full.

Is there anything you would like to ask me about the research or the interview before we start?

Seek additional verbal consent to continue and to audio-record the interview.

# 1: About you

- Please tell me about yourself

# What is your experience of being involved in research?

# Probe for extent of experience, how came to be involved

# Probe for example research projects, identify examples of statistical methodology research

# 2: Values

# Thinking about your experience of statistical methodology research...

# What motivates you to be involved in statistical methodology research?

# What do you enjoy about being involved?

# What do you see as the benefit to the research of involving members of the public?

# 3: Approaches and Methods

# Thinking about a project you have been involved in...

# How were you involved in the research?

# What stages of the research were you involved in?

# In what ways, if any, did you feel able to contribute to the research?

# How did the research team make sure you understood the research and could contribute?

# Probe for strategies or methods used, with examples

# What worked well?

# What did not work well?

# Were there any challenges for you in being involved?

# Probe for challenges around understanding, communication, practical challenges

# 4: Impacts

# Thinking about a current or recent project...

- Have there been opportunities to reflect on how the PPIE is going?

# Has PPIE made a difference to the research?

# Probe for difference made by the PPIE group and by the individual

# Probe for positive and negative impacts

# How do you know this?

# 4: What does good look like?

# How can we make sure that patient and public involvement in statistical methodology research makes a difference?

# What would you like researchers to know about involving patients and the public in their research?

# 5: Closing questions

# Is there something more you would like to share about being involved in research?

# Do you have any questions for me?

**6: Demographic Questions**

Before we finish, I will **switch off the recording** and I would just like to collect some information about you. This information is important so that we know who we have spoken to and who we haven’t spoken to yet. All the questions are optional and you can choose not to answer.

# How would you describe your gender?

# What is your age?

# Under 25 years

# 25-39 years

# 40-59 years

# 60 years and over

# How would you describe your ethnic background?

### Asian or Asian British

- Indian
- Pakistani
- Bangladeshi
- Chinese
- Any other Asian background

### Black, Black British, Caribbean or African

- Caribbean
- African
- Any other Black, Black British, or Caribbean background

### Mixed or multiple ethnic groups

- White and Black Caribbean
- White and Black African
- White and Asian
- Any other Mixed or multiple ethnic background

### White

- English, Welsh, Scottish, Northern Irish or British
- Irish
- Gypsy or Irish Traveller
- Roma
- Any other White background

### Other ethnic group

- Arab
- Any other ethnic group

Is English your first language?

What is your postcode?

What is your highest qualification?

No qualifications

NVQ, BTEC National, OND or ONC, City and Guilds Craft or Advanced Craft

GCSEs or equivalent

AS, A level or equivalent

Degree level or above

*Thank you so much for taking the time to speak with me and contribute to the research*
