# Supplementary material for: How Do We Achieve Impactful Patient and Public Involvement and Engagement in Statistical Methodology Research? A Qualitative Research Protocol
Source: Health Expect. 2026 Aug 6;29(4):e70777. doi: 10.1111/hex.70777 (PMC13445566; doi:10.1111/hex.70777)
Supplement: Supplementary file 3 [file HEX-29-e70777-s003.docx]

# How do we achieve impactful patient and public involvement and engagement in statistical methodology research?

**Topic Guide for research staff**

**Welcome and Introduction**

Description of the project and the focus of the interview.

We are speaking to research staff with experience of involving members of the public in statistical methodology research. By this we mean research about how we conduct statistical research.

You are of course free to pause or stop the interview at any time. If there are any questions that you don’t want to answer, we can miss them out.

We are very mindful of the need to protect relationships between researchers and public contributors and we want you to feel able to speak freely. Your participation will be kept confidential. We may use direct quotations from your interview when we report the findings of the research but we will remove anything that could identify you. We will create composite case studies that will bring together experiences from different people, rather than reporting one person’s experience in full.

Is there anything you would like to ask me about the research or the interview before we start?

Seek additional verbal consent to continue and to audio-record the interview.

# 1: About you and your role

- Please tell me about your academic background and your current role

# 2: Experience and views of public involvement in research

# What is your experience of involving patients and the public in research?

# Probe for length of time and extent of experience

# Probe for any training received

# Probe for example research projects, focus on statistical methodology research

# What do you see as the potential benefits of involving patients and the public in statistical methodology research?

# Probe for with examples

# What are the challenges of involving patients and the public in statistical methodology research?

# Probe for examples

# Probe for comparisons with other types of research, where the participant has relevant experience

# How would you describe your approach to involving patients and the public in your statistical methodology research?

# Probe for strategies or methods used, with examples

# What works well?

# 3: What does good look like?

# What are the characteristics of high quality, impactful PPIE in statistical methodology research?

# Can you give an example of where PPIE has made a tangible difference to your research?

# What support for PPIE is there within your institution?

# Infrastructure, staffing, funding etc.

# What would help you to continue to develop your practice?

# What would early career researchers benefit from to develop skills in PPIE for statistical methodology research?

# 4: Closing questions

# Is there something more you would like to share about involving patients and the public in research?

# Do you have any questions for me?

**5. Demographic Questions**

Before we finish, I will **switch off the recording** and I would just like to collect some information about you. This information is important so that we know who we have spoken to and who we haven’t spoken to yet. All the questions are optional and you can choose not to answer.

# How would you describe your gender?

# How would you describe your ethnic background?

### Asian or Asian British

- Indian
- Pakistani
- Bangladeshi
- Chinese
- Any other Asian background

### Black, Black British, Caribbean or African

- Caribbean
- African
- Any other Black, Black British, or Caribbean background

### Mixed or multiple ethnic groups

- White and Black Caribbean
- White and Black African
- White and Asian
- Any other Mixed or multiple ethnic background

### White

- English, Welsh, Scottish, Northern Irish or British
- Irish
- Gypsy or Irish Traveller
- Roma
- Any other White background

### Other ethnic group

- Arab
- Any other ethnic group

# How would you describe your career stage?

*Thank you so much for taking the time to speak with me and contribute to the research*
